# Life strategies of people with deafblindness due to Usher syndrome type 2a - a qualitative study

**DOI:** 10.1080/17482631.2019.1656790

**Published:** 2019-08-30

**Authors:** Mattias Ehn, Agneta Anderzén-Carlsson, Claes Möller, Moa Wahlqvist

**Affiliations:** a The Swedish Institute for Disability Research, Örebro University, Örebro, Sweden; b University Health Care Research Centre, Faculty of Medicine and Health, Örebro University, Örebro, Sweden; c Audiological research centre,University hospital, Faculty of Medicine and Health, Örebro University, Örebro, Sweden; d The Swedish National Resource Centre for Deafblindness, Lund, Sweden

**Keywords:** Deafblindness, focus groups, life strategies, psychological flexibility, qualitative content analysis, Usher syndrome type 2

## Abstract

**Purpose**: To explore life strategies in people with Usher syndrome type 2a.

**Background**: There are no studies on life strategies in people with Usher syndrome. People with deafblindness are often described in terms of poor health and low quality of life, or as being vulnerable. From a clinical point of view, it is of importance to balance this picture, with an increased knowledge of life strategies.

**Methods**: The study had a qualitative explorative design. Fourteen people aged 20–64 years (4 women, 10 men) with USH2a in Sweden participated in focus group interviews, which were transcribed and analysed by qualitative content analysis.

**Results**: The content analysis resulted in seven categories; *remaining active, using devices, using support, sharing knowledge, appreciating the present, maintaining a positive image* and *alleviating emotional pain*. Two sub-themes: *resolve or prevent challenges* and *comforting oneself* was abstracted *forming a theme “being at the helm”*.

**Conclusion**: The findings show that people with USH2a have a variety of life strategies that can be interpreted as highlighting different aspects of psychological flexibility in a life adjustment process. The study demonstrates that people with USH2a manage in many ways, and metaphorically, by “taking the helm”, they strive to actively navigate towards their own chosen values.

## Introduction

Deafblindness, also known as dual sensory loss, varies in diagnosis, onset, auditory and visual abilities. The Nordic definition of deafblindness states:

“Deafblindness is a combined vision and hearing impairment of such severity that it is hard for the impaired senses to compensate for each other. Thus, deafblindness is a distinct disability. To varying degrees, deafblindness limits activities and restricts full participation in society. It affects social life, communication, access to information, orientation and the ability to move around freely and safely. To help compensate for the combined vision and hearing impairment, especially the tactile sense becomes important …” (Nordic Centre for Welfare and Social Issues, 2018)

The situation of persons with deafblindness has been studied, showing psychological distress, unmet needs and lack of formal support (Bodsworth, Clare, Simblett, & Deafblind, UK 2011). People who become deafblind have been described in terms of interactional powerlessness, vulnerability and struggling hard to adapt in a world that is sometimes perceived as hostile (Schneider, 2006). Stigmatization has been reported in relation to the use of mobility and communication aids (Hersh, 2013b). The situation of persons with deafblindness has also been referred to as a constant ontological insecurity (Danermark & Möller, 2008). Möller (2008) states that the functional limitations in hearing and vision present in deafblindness lead to a vulnerable situation, due to the difficulties in accessing information and in face-to-face interaction with other people. Isolation and social exclusion are a common consequence, as are restrictions in terms of activity and increased risk of physical harm (Möller, 2008).

Despite the challenges described above, a systematic review revealed that persons with deafblindness do not view themselves as permanently vulnerable, but research tends to focus on negative outcomes instead of exploring positive risk taking, coping capacity and resilience (Simcock, 2017). Similarly, Schneider (2006) emphasized that despite their vulnerability, people with deafblindness have a range of strategies to adapt to the challenges inherent in their situation (Schneider, 2006). They have also been found to be interested in being involved and contributing to society (Hersh, 2013a). A scoping review revealed that participation among people with deafblindness is due to the interaction of personal and environmental factors, but called for studies focusing on lived experiences in order to increase understanding and improve services to enhance participation (Jaiswal, Aldersey, Wittich, Mirza, & Finlayson, 2018).

The importance of understanding deafblindness from a process oriented perspective has been emphasized through a life adjustment model (Gullacksen, Göransson, Rönnblom, Koppen, & Rud Jörgensen, 2011). There, the progressive course that is common when living with deafblindness is described as an ongoing process, in which personal and social changes to handle the progression of hearing and vision loss are emphasized (Gullacksen et al., 2011).

Among the syndromes leading to deafblindness, Usher syndrome (USH) is the most common (Möller, 2003). USH is an autosomal recessive inheritance disorder that affects hearing, vision and in some cases vestibular function (Möller, 2003). The estimated prevalence of USH is 6 per 100,000 individuals (Kimberling & Möller, 2013). It can be divided into three clinical subgroups, USH 1–3 (Millan et al., 2011). The clinical sub-groups differ in degree of hearing loss, vision and balance problems and to date, 13 genes have been detected in USH (Mathur & Yang, 2015).

Usher syndrome type 2 (USH2) is characterized by a congenital moderate to severe hearing loss. A retinal disorder, retinitis pigmentosa (RP), causes a progressive vision loss due to retinal degeneration leading to impaired adaptability to light, light sensitivity, night blindness, visual field limitations and impaired visual acuity (Hartong, Berson, & Dryja, 2006). In adulthood, the progression results in severely impaired vision (Kimberling & Möller, 1995). USH 2 is the most common form of USH in most countries (Leijendeckers, Pennings, Snik, Bosman, & Cremers, 2009; Sadeghi, Eriksson, Kimberling, Sjöström, & Möller, 2006). Approximately 72–87% of persons with USH 2 have genetic variations causing Usher type 2a (Baux et al., 2007; Dreyer et al., 2001; Pennings, 2004). The hearing impairment in USH2a has been studied, showing a homogenous pattern (Hartel et al., 2016).

Most studies of the psychosocial situation of those with USH include participants with great differences in hearing loss, balance problems and vision loss. Some use sign-language and others oral communication (Ellis & Hodges, 2013; Evans, 2017; Högner, 2015). Such diversity could pose a threat to conclusive study results. Nevertheless, depression and loneliness have been shown to be common and strongly related to perceived poor quality of life in persons with USH, where the importance of receiving social support has been emphasized (Dean, Orford, Staines, McGee, & Smith, 2017). High levels of stress are common (Högner, 2015). Ellis and Hodges (2013) interviewed people with USH in the UK and their narratives reveal a situation of ongoing change due to the diagnosis and the constant challenge of trying to create predicatiblity in an unpredictable situation. However, they also emphasize the great diversity within the USH group. The informants in their study described increased dependence and problems with the unequal distribution of deafblind-related services (Ellis & Hodges, 2013). In a thesis by Evans (2017), the lived experiences of people with USH focused on the diagnosis, family relationships during the life span, sense of belonging and experiences of professional support. The study highlights that although USH is not life threatening, it is certainly life altering, affecting the whole family. Deafblind culture and tecnology could endow a sense of belonging, but Evans also reveals the urgent need for specialized support and increased awareness among professionals and the public.

To the best of our knowledge, no studies on psychosocial aspects have specifically controlled for genetic variations of USH2a. However, a few studies focused on the situation of persons with USH2, revealing poor physical and psychological health with an increased risk of fatigue and suicidal behaviour (Wahlqvist, Möller, Möller, & Danermark, 2013). A study of perceived independence in USH2 showed that the need of support increases with higher age (Damen, Krabbe, Kilsby, & Mylanus, 2005).

To date, most studies on USH have focused on various problems or challenges and to the best of our knowledge, there is no study that exclusively explored the strategies employed by people with USH to manage life. The clinical subgroups of people with USH differ in genetics, hearing and vision progression and there is a need for studies that focus on the specific situation of various clinical and genetic sub-groups of people with USH, for example people with USH2a. Such specific knowledge is important for the planning of rehabilitation interventions and as a foundation for evidence-based support guidelines. From an ethical perspective, it is important that people with deafblindness, in this study represented by people with USH2a, not only are described as vulnerable, but rather as people with competences and resources.

## Purpose

The aim of the study was to explore life strategies in people with Usher syndrome type 2a.

## Material and methods

### Design

The study had a qualitative explorative design (Patton, 2002) focusing on the participants’ own perspective of their everyday life. Data were collected through focus group interviews (Kitzinger, 1994), and an inductive qualitative content analysis was employed (Graneheim & Lundman, 2004; Krippendorff, 2012).

### Participants and setting

The participants with USH2a were recruited in 2013 from a research register of persons with USH that includes approximately 50% of the total USH 2a population in Sweden. At the time of the study, 58 persons of all ages with a genetically confirmed diagnosis of USH2a had been registered, from whom a purposeful sample was selected. Persons were regarded as eligible if they were of working age (18–65 years) and capable of participating in a focus group using verbal communication. The sampling strived to achieve heterogeneity in terms of age and sex, as well as geographic distribution. Thirty-two persons were invited to participate. An invitation letter in large print was sent by post. The decision to only invite people with USH2a was mainly logistic, as the data collection was conducted at a research event for people with USH2a, where the participants also took part in research focusing on cognitive assessments and were offered social activities.

Of the 32 persons invited, 14 agreed to participate, 5 declined, 1 person initially accepted, but was unable to attend the research event and 12 persons did not respond to the invitation. We have no further information about the non-participants. All 14 individuals (4 women and 10 men) who agreed to participate were included in the study. They ranged in age from 20 to 64 years and lived in different parts of Sweden. All participants travelled to take part in the interviews, which were conducted at an audiological research centre in Sweden. Hearing was assessed at the time of the study and all participants met the criterion of moderate to severe hearing loss. All visual assessments were conducted at different low vision clinics at a time close to the data collection. No study-specific assessments were performed and instead, medical reports including the above-mentioned assessments were retrieved after authorization by the participants. Data on age, hearing and vision are presented in Table I.

**Table I.** Demographic data.

	Demographics	Range
N	14	
Women, n (%)	4 (29%)	
Age mean (SD)	41 y (13 y)	20–64 y
Hearing loss diagnosis age mean (SD)	4 y (2 y)	0,5–10 y
Vision loss diagnosis age mean (SD)	22 y (9 y)	7–42 y
Hearing loss PTA4* mean (SD)	65 dB (9 dB)	48–80 dB
Visual field category** (median)	4 (<10 degrees)	2–5
Best corrected visual acuity*** mean (SD)	0,4 (0,3)	0,05–1,0

*Hearing was assessed by Pure-tone audiometry with calculation of the pure tone average for the frequencies 0.5, 1, 2, and 4 kHz (PTA4). Thresholds were classified from mild to profound hearing loss.

**Visual field tests were performed by Goldmann perimetry and categorized into five phenotypes (1–5): category 1 was a normal visual field; category 2 the presence of a partial or complete ring scotoma (the latter either extending or not extending into the periphery); category 3 concentric central field loss with a remaining peripheral island less than one-half of the field circumference; category 4 marked concentric loss <10 degrees; and category 5, no visual field at all (blind) (Grover, Fishman, Anderson, Alexander, & Derlacki, 1997).

***Best corrected visual acuity (VA) (the ability to discriminate details) was measured by Snellen chart-based standard tests, given in decimals, 1.0 indicating normal VA whereas 0.05 is a severely reduced VA (in the US VA ≤ 0.1 is defined as legal blindness).

### Data collection

Data were collected by means of focus group interviews. Focus group interviews enable the use of group interaction to elicit data, where the participants are encouraged to talk, ask questions and comment on each other’s statements in order to obtain a range of perceptions (Kitzinger, 1994). The interaction between the group members can reveal and develop perceptions and attitudes that would not be found in individual interviews (Jamieson & Williams, 2003).

The participants were divided into three focus groups: One with younger men (21–44 years, n = 5), one with older men (41–61 years, n = 5) and one consisting of women (23–61 years, n = 4). The total sample size limited the variability of the grouping and the three focus groups were based on a balance between homogeneity in sex that could promote open discussion and heterogeneity to allow contrasting opinions (cf. Jamieson & Williams, 2003). Any siblings were separated to make sure that everyone could speak freely and increased confidentiality.

The focus groups were moderated by one of the authors (MW), who made sure that all participants had the vision and hearing prerequisites necessary to participate. The moderator is very familiar with persons with deafblindness and was assisted by a senior researcher experienced in research about deafblindness. The moderator introduced each focus group session by informing about the aim of the study and that she would not direct the interview, only facilitate it with minimal intervention in order to promote the participants’ own initiatives in the dialogue (Kitzinger, 1994). Based on the aim of the study the following questions were posed in order to start the focus group discussion: “How do you cope with your life situation and how do you manage difficulties? If you were not successful in dealing with the situation, in what way would you have liked to do things?” Thereafter, the moderator only asked for clarification when something was unclear and facilitated the participants to take turns to contribute to the discussion by verbally indicating when she noticed that someone wanted to say something, thus helping the group to shift attention towards quieter participants. The focus group interviews were held in Swedish oral language and lasted for about two hours with a 20 minute break. They were digitally filmed and audio recorded. During the interviews, all participants used their hearing aids connected to a hearing loop. The focus group interview room was adjusted to reduce noise and had proper illumination. Furthermore, window blinds were used to minimize glare. The participants had the opportunity to be assisted by a significant person, guide or interpreter during the interview. One of the participants had an interpreter and another chose to have a family member at hand.

### Analysis

The focus group interviews were analysed by qualitative content analysis with the aim of understanding the meaning of the text of the interviews and gaining new insights into the particular phenomenon; i.e., life strategies of persons with deafblindness. An inductive analysis was conducted due to the exploratory design of the study (Krippendorff, 2012). All recordings were transcribed verbatim by an experienced secretary and the transcripts were compared to the recordings. All interviews were listened to and read through several times by the first author in order to obtain a sense of the whole. Thereafter meaning units were identified, condensed and labelled with a code (Graneheim & Lundman, 2004). The codes were also labelled with the individuals’ initials in order to follow the statements of each participant during the analysis. A comparison of the codes was made in order to find similarities and differences, after which they were abstracted to manifest categories and sub-categories. The first author was responsible for the analysis in collaboration with the other authors. All authors actively took part in the coding process during research seminars.

All categories were evaluated in a reflective interpretative process, where all authors moved back and forth between meaning units, codes and categories. When discrepancies in interpretation occurred, they were discussed until consensus was obtained. In addition to the manifest categories, a more abstract, latent thematic content running through the entire dataset was found, resulting in two sub-themes and one theme (Graneheim & Lundman, 2004). The video recordings were not used in the analysis, but could be used if any ambiguity was raised when analysing the text based on the audio recordings, such as turn taking or if needing to analyse a non-verbal behaviour. No member checking or audit trail was conducted.

The authors represent different professions with extensive clinical and research experience that enabled active reflection that was valuable in the data analysis. ME is a clinical psychologist with 15 years of experience of patients with USH, CM is a professor in audiology with 30 years of clinical experience and research in USH, AAC is an RN and associate professor specialized in qualitative methods who has been active in deafblindness research for several years. Finally, MW is a PhD and social worker with experience of working with people with hearing loss, deafness and deafblindness, as well as experience of research on USH and health.

### Ethical considerations

USH 2a is a rare diagnosis and therefore extra consideration of confidentiality is necessary. The recruitment of participants from different parts of Sweden reduced the risk of identification and data that could disclose identities (names, places) were removed from the focusgroup interviews. Prior to the interviews, all participants received verbal and written information about the study, that participation was voluntary and that they had the right to withdraw from the study at any time without giving reasons. All participants signed an informed consent form prior to the focus group interviews.

The study conformed to the criteria of the Helsinki declaration for medical research in human subjects. The study was approved by the Regional Ethics Committee in Uppsala (Nr. 2012/515).

## Results

The data analysis resulted in seven categories and a total of 17 sub-categories that are presented below. Most sub-categories were represented by statements in all three focus groups. However, the sub-category *Business as usual* was only represented by statements of the younger men and the sub-categories *Escapism* and *Hope* were only represented by statements in the two groups of men. Besides the manifest content analysis, two latent sub-themes and a superordinate theme emerged. For an overview of the theme, sub-themes, categories and sub-categories see Table II

**Table II.** Theme, sub-themes categories and sub-categories.

Theme	Sub-themes	Categories	Sub-categories
Being at the helm	Resolving or preventing challenges	Remaining active	Business as usual’
			Adapting activities
			Using memory and attention
		Using devices	Accessibility aids
			Everyday tools
		Using support	Formal support
			Informal support
		Sharing knowledge	Informing in everyday situations
			Educating professionals
	Comforting oneself	Appreciating the present	Seize the moment
			Enjoy meaningful activities
		Maintaining a positive image	Negotiate who I am
			Stand up for myself
			Boost self-confidence
		Alleviating emotional pain	Self-distance
			Escapism
			Hope

The theme, sub-themes and categories illustrated by quotations will be presented below.

### Theme being at the helm

The analysis formed an overarching theme *“Being at the helm”* that emerged in the abstraction process of working through the manifest content of the categories. The theme encompasses the participants’ active, cognitive and emotional striving to be the person in control of life domains that they considered of importance. The theme was divided into two sub-themes *Resolving or preventing challenges* and *Comforting oneself.*

### Sub-theme: resolving or preventing challenges

The first four categories, *Remaining active, Using devices, Using support and Sharing knowledge* formed the sub-theme *Resolving or preventing challenges*, which reflects the participants’ ability to manage practical aspects of their life situation.

#### Remaining active

Being able to maintain an active life was discussed and emphasized by all three groups. The content of this category was divided into *business as usual, adapting activities* and *using memory and attention.*

*Business as usual* was only discussed among the younger men and included not restricting or changing their way of life due to limited vision and hearing. Although the participants were aware of their limitations they had not changed their way of performing activities. The participants valued acting as if there were no restrictions and emphasized that their vision and hearing loss should not change or restrict their ability to be active. One of the participants stated:

I just want to be with my friends. I have said from day one that I will refuse to let it [deafblindness] affect my life and prevent me from living the life I want. So then I just go with the flow, so to speak. And sometimes one just sits there and can hear nothing because the music is so loud. But at the same time one doesn’t want to miss out on anything. (Person 6)

However, one of the older participants in the group of young men considered that he had also acted in accordance with the principle of *business as usual* some years ago, but had gradually changed his mind-set and could no longer ignore the fact that the condition sometimes affected his life.

*Adapting activities* was discussed by all three groups. It entailed practical adaptations, as well as adapting the pace, timing and duration of activities. It often included the use of assistive aids, but most of all a change towards thinking “outside the box”. “*It is about being able to have the imagination to find new ways, not being blind to the fact that there is more than one way … for example you may not need to hammer in nails when you can use a screw driver.”* (Person 14)

The experiences of the participants were at times contradictory. Some highlighted the fact that the fairly slow progression of their condition had given them time to gradually adapt and find new ways to deal with everyday situations. For others, adaptation was sometimes difficult as small changes in situational conditions could make a great difference:

For me it is like a situational disability. In many situations … … I’m just as much a part as everybody else, while in other situations it doesn’t work at all. And finding a balance is very difficult. (Person 3)

Adaptation of working life was important for several of the participants. One participant had decided to change career when he became aware that his previous profession would not be possible in the long term due to visual problems. For others, re-education had opened up the possibility for a positive change of career, thus increasing their ability to remain active. Others tried to achieve a sustainable working life by reducing assignments or limiting visually demanding elements of duties, for example travelling. Self-employment also facilitated remaining active:

I have chosen my job and can even choose the working hours that suit me. After all, I’m my own boss so I can decide that I want to work exactly those hours. And now I want to work from home for a few days. (Person 3)

Devoting a great deal of effort to organizing things in the home enabled an independent and active life at home. By organizing things in an individualized way the perception of being isolated and locked up at home was changed to a feeling of being active at home.

Being able to move around safely and independently was important for remaining active and mentioned by all groups. Many of the participants had adapted their way of moving around, being more careful when walking, such as walking slowly or holding someone’s shoulder, especially in unfamiliar surroundings or in the dark. Public transport was necessary and functioned well for some of the participants who had given up driving and cycling. Among the younger participants cycling was still possible in the daytime or in summer when visual conditions were more optimal: “*But in the summer I cycle as much as I can”* (Person 7). One person highlighted the extreme end-points and complexity of the disability, sometimes being able to cycle and sometimes needing to use a cane for mobility.

The participants described adapting their way of exercising to activities that were less visually demanding. For example, playing football was replaced by training at a gym. However, sometimes it was still possible to perform demanding activities for fun, but not on a competitive level. Yet another adaptation of activities was remaining in contact with old friends, despite no longer being active in the sport they used to take part in together.

Adaptation of activities was described in terms of being selective in the choice of activities and company in order to save energy. “*Then you have sufficient strength to have fun and enjoy yourself. Otherwise you just waste energy to show that you are independent. [Instead] you have to use your energy where it is most needed.”* (Person 2)

Adaptation also included optimizing communication during social events. Some of the participants decided to only see a few friends at a time and others refrained from attending noisy parties, but instead choose to attend quiet events. One participant always tried to approach strenuous activities in as relaxed and calm a manner as possible as it had a positive impact on the visual and hearing perception “*If you make too great an effort, then your sight and hearing become worse, or at least they do in my case. So if I’m relaxed and yes calm I can also hear and see better.”* (Person 3)

### Using memory and attention

Several participants expressed that their vision and hearing limitations were to some extent compensated by different cognitive strategies. Memorizing where objects were located facilitated finding them, while spatial information such as what a specific room looked like or how an outdoor environment was organized enhanced the ability to move around safely. Increased attention when moving about was also emphasized. “*I feel that I have to constantly scan the periphery, because all of a sudden somebody may decide to cross my path and perhaps I’m not aware of it …”* (Person 1)

#### Using devices

This category includes different kinds of devices that the participants described using in their daily life to compensate for vision and hearing problems. The category reveals both the importance of specialized aids and the need to be inventive in using items that are at hand. The devices were divided into *accessibility aids* and *everyday tools.*

*Accessibility aids* were products mainly aimed for use by a person with a disability, for example a white cane, a safety alarm and computer screen reader, as well as visually or auditory accessible signs. All participants mentioned a variety of devices that compensated for their hearing and visual problems, but it was the white cane that gave rise to the most interest in the discussions. The experiences of using the white cane differed; some found it unproblematic, while others considered it more controversial and related to negative emotions. Using it could lead to increased security and at the same time be something unwanted: “*I was mainly thinking about the cane, as sometimes you want it and at other times you don’t even want to see it. But you still have it with you and carry it around and that’s what makes it difficult.”* (Person 6)

Likewise, one of the participants had experienced that the white cane had an impact on how he was treated by the surrounding environment and facilitated obtaining help from others when necessary. Moreover, if their behaviour deviated from the norm the white cane helped to normalize the situation. When travelling abroad it limited the need to explain the disability in a foreign language. One participant suggested new items that could complement the white cane in situations where it could not be used, e.g., a marked bathing cap for use in a swimming pool. Despite the positive experiences of using the white cane, there were also accounts of not using it in one’s own neighbourhood due to risk of meeting an acquaintance who was not aware of the severity of a person’s vision problems. Some only used the cane in demanding situations, such as in the dark, when there was a great deal of snow or when using public transport.

*Everyday products* include any other device that the participants could use to compensate for their vision and hearing loss. Two persons described using an umbrella instead of the white cane to facilitate mobility, not making their visual problems obvious: “*But a tip is to carry an umbrella, I usually do if I don’t want people to notice”* (Person 9)

Smartphones and tablets were commonly used by most participants and easily adapted by means of magnification, adjusted fonts and high contrast modes or by adjusting the auditory settings. The tablets and smartphones facilitated communication, access to information and were also used for playing interactive games. Other everyday tools used were increased illumination and larger computer displays.

The limited vision made it difficult to search for small items that were missing and some described using a box to store important items in a designated place at home. All participants were very positive about the use of everyday devices and described that it enabled them to participate in activities, increased their well-being and at the same time made them feel that they could do things in just the same way as anyone else.

#### Using support

This category describes the experiences of using different kinds of support provided by others. The support was both *formal*, given by different agents in society and *informal* when received from family, friends and others.

The participants had used the *formal support* from healthcare providers, including low vision or audiological clinics, and some had also received services from specialized counselling and support teams for persons with deafblindness. Formal support also included doctor’s certificates informing authorities about their diagnosis and disability when applying for social services. Although formal support from the social services (for example sick-leave, disability pension, assistive aids and professional guides) was highly valued, it was at times difficult to obtain. The participants thus needed assistance to apply for services, or to lodge an appeal against refusals from various social services. Thus, having regular contact with the healthcare system was regarded as helpful. Such contact also provided the participants with information about the disease, which was valued. To understand genetics for future family planning was regarded as supportive by some. One of the participants had benefited from psychiatric services but urged that it should not be associated with stigma.

To emotionally connect with the professionals providing personal support was described as important by some. One of the participants especially appreciated the dedicated professionals in the specialized counselling and support teams who not only provided services, but also had an interest in the entire person and sometimes just popped in for a chat.

The personal chemistry was more pronounced when receiving personal support outside healthcare situations. One of the participants had good experiences of one guide, but another guide did not work at all. Others had only positive experiences of receiving support and preferred a guide instead of their spouse when going shopping for clothes. One participant described a positive first experience: *“The first time I got this guide from [name of a town] to accompany me, it was great fun. I could relax”*. (Person 4)

Formal personal support such as a guide increased independence and safety, thus reducing the exposure to demanding situations or avoidance.

I can be independent when I have a guide or interpreter or whatever, then I’m independent. But if I don’t have one and want to go shopping then I become dependent on finding some shop assistant who’ll help me. And that’s when I’m not independent. (Person 3)

Receiving support was described as sometimes natural, yet sometimes challenging. For one of the participants, requesting formal support in the form of a guide was perceived as difficult for reasons of integrity. Although needs would be identified, the risk that everyone in the small village would know about them, with the risk of increased stigmatization, prevented this participant from applying. The experience of using a deafblind interpreter could also be situational; it was described as positive at a healthcare visit, but more complex in informal situations, such as at a party where the interpreter was too much in focus for the participant to feel comfortable.

*Informal support* was support provided by friends or family members. Many of the participants knew that spouses, parents or children were persons who would always stand by their side. The participants had differing experiences of receiving support from friends. The importance of having friends who were aware of the consequences of deafblindness was emphasized. Support, such as holding someone’s shoulder, could facilitate orientation in the dark and a friend driving the participant’s car increased mobility. For some of the younger participants, going to a club, travelling and taking part in sporting events was natural and facilitated by the company of friends, thus regarded as no big issue.

However, being dependent on help from friends was not always easy. Repeated offers of support could be experienced as intrusive and although the participants were aware of their need for assistance, receiving support reminded them of their lost independence. One participant described the ambivalence towards support as follows: “*I feel like a queen sitting there waiting for my husband to bring me the food. You have to try to turn it into something positive. But it is a bit difficult.”* (Person 4)

One strategy for dealing with one’s own functional deficits was to search for other persons with USH who could serve as role models. Such a person was good to talk to, as they shared the same experiences. For some, participation in the focus group was the first occasion on which they met others with the same diagnosis and they were very interested in creating a network to share experiences.

#### Sharing knowledge

The category sharing knowledge reveals an almost never ending need for informing others about USH and its consequences in order to make life easier for themselves. The content was divided into *informing in everyday situations* and *educating professionals.*

*Informing in everyday situation*s included sharing their knowledge about their diagnosis or needs to family and friends. All participants stressed the importance of informing, although at times it was frustrating and emotionally demanding, especially when repeated information seemed to make no difference with regard to adapting behaviour or encounters. Others reported having no problem with repeatedly giving a short description of their condition. As the hearing and vision problems were often invisible to those in the participants’ surroundings, taking the initiative to share information had become even more important as the condition worsened. One participant remarked: “*It is clear that you have to [inform], as it makes it easier for others to comprehend if told how to handle it [deafblindness]. It is not easy for them to understand, because it doesn’t show.”* (Person 3)

The experience was that early information could limit the risk of misunderstandings and increase the possibility of a helping hand. However, information was not equally shared with everyone. One of the participants pointed out the importance of being able to differentiate between levels of narratives appropriate for the given situation, duration and proximity:

… I have maybe three or four really close friends to whom I can tell everything. So I suppose I have told them … … but you may not bother to tell people who you might meet once a month or so … … It depends on when or where you meet them … But of course I tell those who are closest to me. (Person 5)

Openness to the family, especially one’s own children, was highlighted, as it made deafblindness uncontroversial and a natural part of family life. The children then learned to adjust their communication. “*My youngest daughter usually tells me that here comes somebody called so and so, then there is no problem whatsoever”*. (Person 4). However, even children sometimes forget and then more concrete “informative” actions were a helpful strategy:

My children have always [said], for instance when their friends visit them, do not leave your shoes like that because mum will trip over them It has always been like that, if they are thrown in a heap, then they will be thrown out the door. (Person 3)

Although informing had many benefits, it could take a great deal of energy, something the participants did not always have. In such cases the strategies varied; some stressed the importance of always being polite and informative to everyone. Another participant appreciated the support of a friend who told others about his condition after receiving the relevant information, while yet another participant stressed the importance of not leaving the responsibility for informing to anyone else in order to ensure the accuracy of the information. Another tentative strategy was to ask the local newspaper to conduct an interview and report about what it is like to live with deafblindness, as a means of increasing public awareness. Practicing informing others about USH made it easier to know what to say in different situations and led to increased confidence.

Two of the older participants revealed their experiences of the symbolic effect of the white cane in indicating their situation instead of actually telling people about their visual problems. One of them stated: *“I’m using my white cane now. I have started using it more and more this last year. It is my way of telling others that I cannot see … I bring it to make others aware”* (Person 11).

#### Educating professionals

The participants had experiences of meeting professionals who had no, or only very limited knowledge about their condition. The experience of educating those who were assigned to help them was very mixed, where the participants in greatest need of expertise found that the only expert they could rely on was themselves. One of the participants exemplified the never-ending need to educate professionals: *“Then after six or twelve months there is a new official. Which means that you have to educate an endless number of people. And it is the same when you meet doctors. It is so bloody hard”*. (Person 2). The lack of professional continuity within the healthcare system and among the authorities had made one of the participants think about making a power–point presentation to show to each new professional they encountered.

### Sub-theme comforting oneself

The categories *Appreciating the present, Maintaining a positive image* and *Alleviating emotional pain* represent different sides of the sub-theme *Comforting oneself* that reflects the emotional aspects of the participants’ struggle to manage their life situation.

#### Appreciating the present

The category *appreciating the present* includes the participants’ way of handling an uncertain future. Regardless of what the future might bring, the participants had different strategies that helped them to focus on their present situation and not be governed by the future. The category encompasses the ability to *Seize the moment* and the importance of being able to *Enjoy meaningful activities*

#### Seizing the moment

Some of the participants, especially younger persons, stressed the importance of not focusing on the diagnosis and its future consequences, but instead concentrating on positive moments in the present. They described an attitude of taking one thing at the time, handling problems when they occur and not allowing the condition to rule their thoughts. Some hoped that research might come up with a cure in the future, but meanwhile they continued to live a good life.

And today there is gene therapy, as well as a lot of talk about stem cells and such stuff. But I have thought about it for more than 30 years and time just passes by [without a cure]. You have to enjoy life anyway. In my opinion, life must be good, or it’s not worth living. (Person 13)

Fulfilling one’s wishes instead of postponing them was described as important. One such example was learning to drive while being aware of only being able to drive for a few years, but prioritizing it for as long as possible: “*So I feel that you have to do what you are capable of when you are capable of it, then you get the most out of activities you want to do. That’s what I believe anyway. “*(Person 1)

Besides fulfilling wishes, the importance of appreciating small things in life was stressed and also to try to live in the here and now. Small things could be getting up in the morning and seizing the day, enjoying the beautiful weather, a cup of coffee, or a micro pause with a spoon of honey when energy levels were low. Another example was lighting a fire in the fireplace. One participant said:

It is like when I share a bath with my daughter, then we light a lot of candles, which she really loves. And then you relax and sort of feel content and in the here and now. Because then you are blissfully unaware of your problems, even though they still exist. (Person 2)

#### Enjoying meaningful activities

All participants highlighted activities as an important factor for their well-being. For some, working life was of importance both in terms of performance and for the opportunity of job-related social networks. Others stressed the importance of leisure activities for their well-being, such as drawing, painting, reading or playing an instrument. The importance of discovering something new when forced to refrain from familiar leisure activities and the sustainability of the new choices were highlighted. Spending time gardening had generated positive attention from neighbours, which increased their self-esteem. Some of the younger men stressed the importance of exercising or spending time after work having coffee with friends to relax and boost their energy levels. Playing computer games and watching films were also ways to relax or distract themselves from undesired thoughts. One of the participants concluded:

One important thing that becomes clear here [in the discussion] is that if you manage to do something, whatever it is, it gives you a sense of satisfaction, no matter how nice or unattractive the result turns out. It sort of refreshes me” (person 10)

#### Maintaining a positive image

The category *maintaining a positive image* includes aspects such as self-confidence and identity despite the challenges related to the diagnosis. The category was divided into *Negotiating who I am, Standing up for myself* and *Boosting self-confidence.*

*Negotiating who I am* outlines how the participants strived to keep their identity and not be defined by a progressive disease. Strategies for how to keep the initiative, remain independent and accept the consequences of the diagnosis were discussed. The younger men expressed that the hearing impairment was something they had always lived with and accepted, but they did not identify themselves as being deafblind.

My identity … first and foremost I have always had impaired hearing and not deafblindness … I have my interests and yours is music and so on. … I guess it is our identities and our way of spending time together with our friends. Then it is made more difficult by the fact that we have disabilities that are specific to the situation (Person 8)

Some of the participants reported that they had no contact with others who have a hearing impairment or deafblindness because they did not want to identify themselves with this group. Those who were more affected by their pronounced visual symptoms had accepted their diagnosis, but not the future deterioration. “*There are different kinds of acceptance. I accept the fact that I have Usher 2. But I still find it difficult to accept that my vision will deteriorate”* (Person 10)

Another aspect of negotiating the self was to show themselves and others that they could manage their everyday life. Some of the participants stated that being able to perform activities as good as, or even better, than anyone else was of importance for how they viewed themselves: *“I can manage myself, and then one places extremely high demands on oneself. I mean, I place much higher demands on myself compared to other people.”* (Person 2)

For some, being independent was an important aspect of how they viewed themselves, thus revealing the challenges involved in balancing an increased need of help from others and at the same time trying to remain independent: *“Because it is a huge struggle trying to find strategies for living as independently as possible when having a visual impairment. It´s not easy. It’s terribly difficult. And then when you have become used to managing on your own it is difficult to know when you can or should ask for help.”* (Person 3)

One of the participants who had children highlighted the importance of striving to be a good parent despite the disability: *“Of course I have to try to be a good mum, despite my impairments, just like every other mum. I don’t want to be less capable than anybody else. I do my best.”* (Person 4). Other participants planned to have children in the near future and had an ongoing discussion with their partner about how they would be able to take full responsibility for raising children and wanted to be considered a good parent despite their limitations in vision and hearing.

##### Standing up for myself

The participants encountered a variety of challenging situations where they did not only have to solve problems in a different way to others, but also needed to stand up for themselves. Standing up for oneself was closely related to the participants’ perception of themselves in relation to their diagnosis.

Finding one’s own way, not thinking too much about what others think or do and realizing that life does not have to be lived in exactly the same way as everyone else was described as helpful. The participants had to overcome feelings of shame when they encountered persons who pitied them. One participant emphasized not regarding oneself as a victim and was proud of never taking advantage of the diagnosis. Another participant had gradually learned to no longer blame herself for setbacks, but instead to attribute them to circumstances beyond her control and to finally stand up for her need of assistance.

There is no reason to feel ashamed about being in need of some help. I say you must not feel sorry for me … I said it is not me who is the problem, it is my eyesight that is so trying. Because we [those with deafblindness] are just as capable as everybody else, we just have our own prerequisites. (Person 4)

Fighting for their right to services and eventually being successful in appeals despite initial rejections was one way of standing up for oneself and one’s needs:

But it is the lack of knowledge, lack of understanding and the unwillingness that you are up against all the time. And it is really very frustrating when you have to do it yourself, submitting appeals and things like that. (Person 2)

This could strengthen the participants in the continuing strive to achieve their own goals. Furthermore, raising one´s voice at the authorities could be a way to cope with the inequalities caused by a lack of competence. However, sometimes situations came to a point where frustration was expressed as: *“You just want to tell them to get lost …”* (Person 7). However, to only attribute problems to the environment could sometimes delay the process of personal development:

What you are saying now, NN, is that it is easier to blame somebody else or some item. That bloody chair, why is it standing in my way? Then you need not confront yourself. You get the aggression out of your system by swearing a bit. But it is part of the process, sort of. I recognise myself in it and have done exactly the same. (Person 2)

##### Boosting self-confidence

For some of the participants the positive self-image could be boosted by external attributes. For example, two of the participants agreed that they felt more confident when they were well dressed: *“When I dress up … … when I am going out and wear some nice clothes. It is a feeling that one has managed to make one’s life a bit better* (Person 13) … *I agree with you.”* (Person 10).

Driving a car was also of symbolic importance and associated with feelings of confidence. Keeping the car or driving license after being obliged to quit driving was important for some:

Today, being able to get a driving license is a privilege. … … I have retained mine and use it as an identity card. I suppose I should have handed it in but I keep it for my own sake because it feels good to have it. And it is important for my own self-esteem. (Person 2)

#### Alleviating emotional pain

The category *Alleviating emotional pain* comprises the participants’ way of protecting themselves from intrusive thoughts and emotions related to situations in the present or the future. It includes the participants’ *self-distancing* ability, but also encompasses different aspects of *escapism* and the importance of *hope*.

*Self-distancing*, which involves trying to look at oneself, the environment or a situation from a distance and sometimes viewing it from a humoristic angle was a strategy for coping with difficult situations and reducing emotional pain:*“For me, laughing is very important, I mean seeing things in a different light … Some situations may not be a laughing matter but you still laugh.”* (Person 10). One of the participants could see the fun in “having a very close relationship to road signs” [bumping in to them] due to visual problems, which had resulted in being given a humorous nickname. Humour was also used to distance oneself from being emotionally affected by authorities. One participant told about a humorous episode with an ignorant insurance official who went on asking stupid questions. Instead of getting angry, the participant could laugh at the ignorant behaviour.

#### Escapism

Both groups of men discussed how they escaped from thinking about deafblindness. Some of the younger participants had developed good strategies for preventing negative thoughts and emotions. They simply avoided situations where deafblindness was in focus and did not spend more time than necessary talking about it. Instead, they looked for friends in order to have fun. Different activities were helpful for distracting them and preventing negative thoughts: *“I am also quite good at switching off. … … Then I let go, watch a film or play a TV game or whatever. That’s my way of relaxing. I just switch off*. (Person 6)

Another way to ease pain by means of the strategy of escapism was to avoid old friends in order not to be reminded about their previous life: *“It is rather that I distanced myself, not wishing to be in contact with my old friends. They remind me of my previous life, so to speak.”* (Person 11). Symptoms could also partially be reduced by anxiolytic medication and sleeping pills, and sometimes alcohol was used as means to escape from the negative emotions.

*Hope* for the future was discussed by both groups of men and included wishes for a possible cure of the disease in the future and preparations to handle or reduce the uncertainty. A younger participant had thought about the risk that he could become blind in the future but hoped that his vision loss would not deteriorate to that extent: *“I don’t think it will happen to me … … I hope that my health and my body will remain.”* (Person 7). Trying not to worry about the future was a way of not confronting negative emotions.

## Discussion

The aim of the study was to explore life strategies from the perspective of persons with USH 2a. The content analysis resulted in seven categories, where the categories *Appreciating the present, Maintaining a positive self-image* and *Alleviating emotional pain* formed an emotion-focused sub-theme, *Comforting oneself*, while *Remaining active, Using devices, Using support and Sharing knowledge* constituted a practically-focused sub-theme *Resolving or preventing challenges*. During the process of working through the content of the categories a common overarching theme emerged: *Being at the helm*. Below, the results will be discussed in relation to previous literature and from a theoretical perspective.

One way to discuss the life strategies of people with deaf blindness from a psychological perspective is to apply the psychological flexibility model (Hayes, Strosahl, & Wilson, 2012). Psychological flexibility (PF) embraces a variety of human abilities that facilitate identifying and adapting to situational demands. Behaviour or mind-sets need to shift when social or personal functioning is compromised. Balance in important life domains is maintained by awareness, openness and commitment to behaviours in line with deeply held values (Kashdan & Rottenberg, 2010). PF has been described as a process of being fully in contact with the present moment as a conscious human being and maintaining or changing behaviour in line with one´s own chosen values (Hayes et al., 2012). PF entails six core flexibility/inflexibility processes:

- *Cognitive defusion*: the ability to change one’s relation to unwanted thoughts
- *Acceptance*: the ability to embrace private events without experiential avoidance
- *Being present* the ability to be in contact with the present moment
- *Self as context*: the ability to experience events and be able to separate experiences from content
- *Value*: qualities and desires that can be manifested in purposive action
- *Committed action*: An ongoing increase in effective behaviour patterns towards chosen values (Hayes et al., 2012).

The six interrelated core processes form the two aspects of PF, commitment-behaviour activating processes and mindfulness-acceptance processes (Hayes et al., 2012). These aspects are similar to the two sub-themes identified in the present study. The first sub-theme in our study, *Resolving or preventing challenges*, has similarities to the commitment and behaviour activating aspect and the second sub-theme *Comforting oneself*, relates to the latter aspect of mindfulness and acceptance.

The two categories *Remaining active* and *Appreciating the present* both comprises different, sometimes overlapping, perspectives on the participants’ experiences of various activities of importance for the well-being. The participants enjoyed their activities from a goal-orientated perspective, for the pleasure of performing and for social well-being. The health related importance of employment has previously been studied in USH2, where employed people exhibited significantly better health than those who had a disability pension (Ehn, Möller, Danermark, & Möller, 2016). The negative effects of long-term sick leave have similarly been shown by Ellis and Hodges (2013). Our participants, especially those who were no longer in paid employment, stressed the importance of meaningful leisure activities. No studies have previously focused on the importance of leisure activities for persons with USH. The psychological flexibility model (Hayes et al., 2012), as mentioned above, stresses the importance of activity and commitment to one’s values for growth and well-being. In line with this, our results show that the participants highly valued being active, where maintaining working-life and leisure activities seems to play an important role. To obtain the value of being active, the participants described continuing in line with business as usual on some occasions, but in many cases they needed to adapt the activities. Some adaptations can be interpreted as more behaviour oriented, while others are related to the use of memory and attention. Cognitive and behaviour adaptation ability can be interpreted in terms of committed action with an ongoing expansion of effective behaviours (cf. Hayes et al., 2012). The results showed that younger participants often acted in accordance with *business as usual*, continuing to do things in the same way as always despite having USH, as compared to the somewhat older persons. This difference could partly be explained by the progressive vision loss of USH, where younger people have a less pronounced visual problem and can remain active without any adaptations (Mathur & Yang, 2015).

Being able to appreciate the present rather than focusing on an uncertain future was helpful for the participants. Similar strategies have been shown to be successful among people with USH in Great Britain (Ellis & Hodges, 2013), where interviewees reported handling their diagnosis by focusing on the present. The participants’ ability to appreciate concrete experiences in the here and now seems to protect them when managing situations that have been described in other deafblind-related studies as characterized by a lack of predictability associated with ontological insecurity (Danermark & Möller, 2008). Acceptance and mindfulness processes as a means to achieve contact with the present, instead of being occupied by the past or a feared future, have been associated with psychological flexibility and well-being (Hayes et al., 2012).

The use of support entailed aspects such as the complexity of how to accept the support required, and the focus group discussions revealed that using support and sharing knowledge was something the participants gradually learned to handle. The process of finding one’s own way included processes of inner negotiation and at times standing up for oneself. The process of using support indicates overlapping psychological flexibility processes of committed action and acceptance but also exemplifies that psychological flexibility is a process that occurs in a social context (cf. Hayes et al., 2012). However, the process of using support was delicate, especially in situations where the participants had finally overcome their own inner conflict about accepting support, only to find the authorities questioning their need for it. Research has shown that lack of support entails a significant risk of loneliness and low quality of life among people with USH (Dean et al., 2017). At the same time, increasing levels of social support predicted high levels of mental health-related quality of life (Dean et al., 2017). The lack of and unequal distribution of support among people with USH is unfortunately one of the most common themes reported in research on persons with USH (e.g., Dean et al., 2017; Ellis & Hodges, 2013; Evans, 2017; Schneider, 2006).

The use of devices was something that the participants discussed. However, it is interesting to note that no one talked about hearing aids, although all fourteen participants were hearing aid users. This might be due to the participants’ familiarity with and well-being in a stable hearing situation. Devices related to the progressive visual impairment evoked more attention, indicating the participants’ need for visual compensation adjustments. The white cane was focused on and some, especially younger participants, limited their use of the cane, or did not use it at all. Sometimes this was because it was not expedient, but on other occasions, it was to avoid potentially negative social consequences. However, older participants who had frequently used the cane revealed only positive experiences. The ambivalence concerning the cane has previously been reported by Ellis and Hodges (2013) and Hersh (2013b), who found that people with deafblindness often perceive the white cane as stigmatizing. The discussion among the participants showed the importance of finding one’s own way to approach controversial accessibility aids. Some participants revealed that by using an umbrella they had found a way to avail of the benefits of an accessibility aid without disclosing their visual impairment. Similar “umbrella-compromises” were also found by Ellis and Hodges (2013). The importance of adjustable everyday devices cannot be underestimated, as they not only practically facilitate the participants’ lives, but also help them to gradually approach situations with a reduced risk of unwanted emotions and a preserved self-image. This can be interpreted as an elaboration of behaviour activation towards important values, where emotional avoidance still prevents a more pronounced change in behaviour that would call for a higher degree of acceptance (cf. Hayes et al., 2012).

Balancing activity with limited energy was something the participants regarded as important. Wahlqvist (2013) has shown that among people with USH2 fatigue was by far the most reported health problem. The narratives of the participants in our study reveal a process of committed action in terms of selecting activities, which was sometimes facilitated by acceptance processes and restricted by denial or avoidance. This life adjustment process among people with progressive deafblindness has previously been described by Gullacksen et al., (2011). Gullacksen’s life adjustment model illustrates the process of initial denial, where people with deafblindness tend to hang on to old strategies. At a certain point in life they have to recognize their situation and thereafter gradually explore and become more rooted in themselves in order to live a good life (Gullacksen et al., 2011). The categories *maintaining a positive self-image* and *alleviating emotional pain* indicate a process where the participants try to accept, or sometimes avoid experiencing, their condition. This is done by means of a stepwise inner negotiation and striving to receive recognition from others by standing up for oneself. The process also shows more avoidant sides, i.e., escapism that could be interpreted in terms of psychological inflexibility (cf. Hayes et al., 2012). However, avoidant emotional coping has been shown to be a strategy that at least in a short-term perspective can be regarded as protective when more problem focused strategies are not at hand (Lazarus, 2006). More adaptive strategies such as self-distancing in the form of humour were also revealed. Humour has previously been shown to be an effective way for people with USH to cope with stressful situations (Högner, 2016) and can be interpreted as an example of cognitive defusion as a means of acceptance (cf. Hayes et al., 2012).

## Strengths and limitations

The qualitative explorative design of the study was regarded as appropriate in view of the fact that there was no previous research on life strategies among people with USH2a. To the best of our knowledge, this is the first focus group interview study with people with USH2a. The use of focus group interviews enabled interaction between the participants that elicited a range of perceptions. The trustworthiness of the study was enhanced through audio and video recording, as well as professional transcription. Furthermore, the involvement of all authors in the content analysis strengthened the credibility and confirmability (Graneheim & Lundman, 2004). The trustworthiness was increased by all participants having a genetically verified USH2a diagnosis and that the background hearing and vision data were not only self-reported. The group of persons with USH2a is relatively small and ensuring the attendance of persons of different ages and gender from various parts of Sweden was challenging. Thus, one limitation of the study is the small number of women who participated. The ability to assess transferability to other groups of people with deafblindness is enhanced through the rich presentation of results and back-ground data (Graneheim & Lundman, 2004). As USH2a represents about 70–80% of the total population of persons with USH2 and as other genetic forms of USH2 involve similar vision and hearing problems, it is likely that the results are representative of people with USH2.

## Conclusion

Our results show that people with USH2a have a variety of life strategies to handle deafblindness-related challenges with a high degree of psychological flexibility. This contradicts the common description of people with deafblindness as vulnerable. Our results show that by *Being at the helm* the participants are committed agents in a process of striving to live an active life in accordance with their own values.

## Clinical implications and future research

The experiences shared by our participants could be used by professionals as an inspiration or starting point to draw attention to important life areas that might need to be addressed in counselling. Furthermore, the findings could be used to identify areas of psychological inflexibility that need attention, but just as important, they could also help people with USH2 to recognize their own personal resources and to emphasize psychological flexibility. The findings underline the importance of being offered early intervention as well as psychological, medical and technical support.

The findings could also form a base for designing research exploring life strategies in other clinical or genetic sub-groups of USH to attain a more comprehensive picture of the experiences of living with USH. Future studies concerning how to support people with USH2 to continue striving towards important values in life are also of significance.
